# Effectiveness and Safety of Preoperative Oral Carbohydrates in Enhanced Recovery after Surgery Protocols for Patients with Diabetes Mellitus: A Systematic Review

**DOI:** 10.1155/2020/5623596

**Published:** 2020-02-18

**Authors:** Li-Na Ge, Lin Wang, Feng Wang

**Affiliations:** ^1^Department of Obstetrics and Gynecology, Shengjing Hospital of China Medical University, Shenyang, China; ^2^Department of Orthopaedics, The First Affiliated Hospital of China Medical University, Shenyang, China

## Abstract

To evaluate the necessity and safety of preoperative oral carbohydrates in enhanced recovery after surgery (ERAS) protocols for diabetes mellitus patients. We searched PubMed, EMBASE, the Cochrane Library, Chinese Biomedical Literature Database, China National Knowledge Infrastructure, and WANFANG databases for articles published through September 2018. We used the Cochrane risk-of-bias tool to assess the methodological quality of included studies. Literature screening, data extraction, and quality evaluation were performed independently by two investigators. Of the 6328 retrieved articles, five eligible randomized controlled trials were included. Two were from China and three were from Germany, Sweden, and Canada. Preoperative oral carbohydrates may facilitate control of preoperative blood glucose, improve postoperative insulin resistance in diabetes patients, and decrease the occurrence of adverse reactions. However, the overall quality of the included studies was low. The available evidence shows that preoperative oral carbohydrates are probably beneficial for patients with diabetes mellitus. High-quality, large randomized controlled trials are needed to verify our findings and provide quantitative results.

## 1. Introduction

Enhanced recovery after surgery (ERAS) perioperative protocols include changes in the conventional preoperative fasting practices and are supported by evidence-based medicine that preoperative carbohydrates reduce surgical trauma and stress, promote early eating and activity of patients, and shorten the recovery time. Carbohydrates intake 2-3 hours before surgery can reduce thirst, hunger, tension and anxiety, insulin resistance, nausea, vomiting, and other complications [1]. The benefits of ERAS guidelines that recommend carbohydrate intake 2-3 hours before surgery for colorectal surgery, gastrectomy, pelvic surgery, and gynecological surgery are recognized [2–5]. More than 15% of general surgery patients have diabetes mellitus [6]. ERAS guidelines do not address those patients, and there are no widely accepted indicators of the effects of preoperative oral carbohydrates in surgical patients with diabetes mellitus. The objective of this analysis is the systematic evaluation of the necessity and safety of preoperative oral carbohydrates for patients with diabetes mellitus. The aim is to provide a basis for the development of ERAS pathways for diabetes patients.

## 2. Materials and Methods

### 2.1. Search Strategy

We searched PubMed, EMBASE, the Cochrane Library, Web of Science, Chinese Biomedical Literature Database, China National Knowledge Infrastructure, and WANFANG databases for publications through September 2018. The search terms were (enhanced recovery after surgery OR ERAS OR surgery OR preoperative OR perioperative) AND (CHO OR glucose drink OR carbohydrate loading OR glucose OR carbohydrates) AND (diabetes mellitus, type 2 OR diabetes OR diabetic hyperglycemia OR glucose intolerance OR T2DM). The reference lists of the retrieved articles were checked for additional relevant studies.

### 2.2. Inclusion and Exclusion Criteria

Two authors independently performed the search, deleted duplicate records, screened the titles and abstracts for relevance, and identified records for inclusion or exclusion. In case of uncertainty, the full-text article was reviewed for eligibility. Disagreements were resolved by discussion and consensus. Clinical trials including surgical patients with type 2 diabetes mellitus, oral carbohydrates or sugary beverages 2-3 hours before surgery, and no study limits on the quantity and variety, a control group without preoperative oral carbohydrate or with a placebo, and study outcomes that included perioperative blood glucose concentration, insulin resistance index, preoperative duration of gastric emptying, duration of postoperative hospital stay, and the incidence of complications were eligible. Reviews, abstracts, animal studies, studies with incomplete experimental data, and duplicate publications were excluded.

### 2.3. Data Extraction and Study Quality

The first author, year of publication, study population, comparison group, and outcome characteristics were extracted by L-NG and independently reviewed by LW. The study characteristics were entered on Excel worksheets (Microsoft Corporation, Seattle, WA, USA).

The Cochrane risk-of-bias tool was used to evaluate the included studies. The study quality evaluation included the randomization method and concealment; blinding of study investigators, evaluators, and participants; integrity of outcome indexes, and selection and other study biases. The studies were rated as having low, high, and uncertain risk of bias. Low risk of bias was required to achieve high quality.

## 3. Results

### 3.1. Study Selection

**Figure 1:**
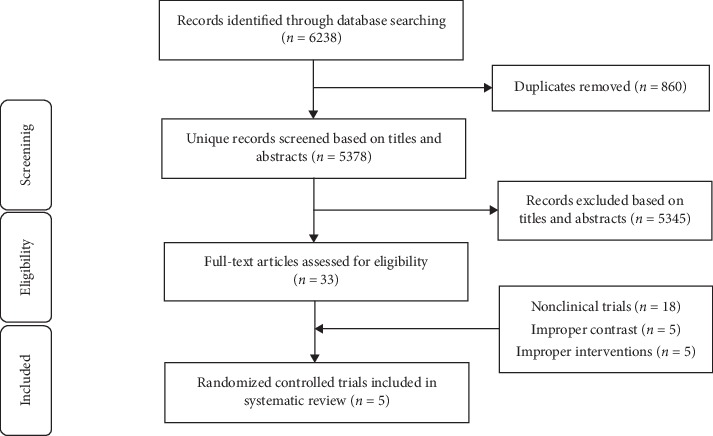
Flow diagram of the study selection process.

The screening process is shown in Figure 1. We retrieved a total of 6238 articles, and after removing duplicates, 5345 were excluded following review of the title and abstract. The full text of the remaining 33 articles was screened, 18 were excluded because of the study design, and ten were excluded because of inappropriate comparisons or interventions. The remaining five studies were included in the review.

### 3.2. Study Characteristics and Quality Assessment

The characteristics of the included studies are summarized in Table 1. All five were trials published between 2006 and 2018. Two were from China and three were from Germany, Sweden, and Canada. One was a randomized trial [7]. Four were prospective observational cohort trials that included patients with type 2 diabetes mellitus [8–10] or both type 1 and type 2 diabetes patients [11]. Four studies had blank, placebo, and/or intervention controls [8–11], and one was placebo-controlled and blinded to both investigators and participants [7]. Three evaluated the occurrence of postoperative complications without finding significant differences between presence or absence of preoperative carbohydrates (*P* > 0.05) [7, 9, 11].

**Figure 2:**
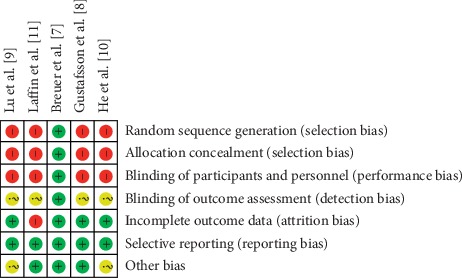
The risk-of-bias summary of the included studies.

**Figure 3:**
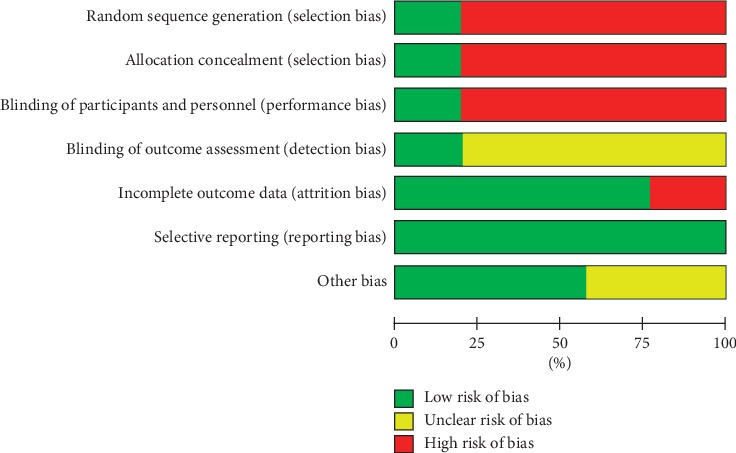
The risk-of-bias assessment of the included studies.

Two trials used postoperative insulin resistance as a biochemical indictor of the effectiveness and safety of preoperative oral carbohydrates for patients with diabetes mellitus [7, 9]. Breuer et al. did not find a significant difference in the insulin resistances of the intervention group and the controls (*P* > 0.05) [7]. Lu et al. reported that, after surgery, the insulin resistance index of the intervention group (3.24 ± 1.07) was significantly lower than of controls (7.40 ± 3.25, *P* < 0.05). Four studies monitored blood glucose in surgical patients [8–11]. One study [8] showed that the peak blood sugar of surgical candidates with diabetes were higher than that of surgical candidates without diabetes after intake but returned to normal values within 3 hours (*P* < 0.05). Two studies [9, 11] found no significant differences in preoperative blood sugar in the intervention and the control groups (*P* > 0.05). One study reported that fasting plasma glucose was lower on the day after surgery in the patients who had been given preoperative carbohydrates than in control patients who had not (*P* < 0.05) [10]. Without blinding, study participants are aware of their group assignment, which leads to inclusion bias. Bias risk maps and summaries are shown in Figures 2 and 3.

## 4. Discussion

The systematic review found that preoperative oral carbohydrate is effective, safe, and feasible for diabetes mellitus patients. Carbohydrate intake 2-3 hours before surgery can avoid hypoglycemic reactions caused by perioperative fasting, decrease thirst and hunger before surgery, and reduce excess secretion of gastric juice and delayed gastric emptying, aspiration, pneumonia, and other adverse events. Although oral carbohydrates before surgery may increase blood sugar for a short time in diabetes mellitus patients, there is no demonstrated risk of hyperglycemia or adverse effects on surgery. Breuer et al. reported that preoperative carbohydrate intake did not improve insulin resistance in diabetes patients after surgery [7]. Lu et al. did observe a decrease in the insulin resistance index of diabetes patients, given 200 mL of a 5% glucose solution 2-3 hours before surgery compared with control patients [9], which was consistent with the findings of Jodlowski et al. [12]. The evidence of the included studies shows that oral carbohydrates 2-3 hours before surgery was safe and tolerable in diabetes patients and had both perioperative and postoperative health and metabolic benefits and improved postoperative insulin resistance. Two studies investigated whether preoperative carbohydrates improved the discomfort in diabetes patients that resulted from fasting and abstinence [7, 9]. One found no effect on preoperative discomfort study [7]. The second study reported decreased preoperative thirst and hunger but no significant effect on preoperative anxiety. There is a lack of consensus that preoperative intake of carbohydrates may alleviate preoperative discomfort in diabetes patients, but the evidence does indicate that it did not cause discomfort.

The lack of glycogen reserve in fasting diabetes patients can lead to an insufficient energy supply, strong fluctuation of blood glucose, increased risk of hypoglycemia, microvascular, nerve, and kidney damage, injury of other tissues and organs, and the occurrence of various acute and chronic complications [13]. In accordance with the British guidelines for perioperative management of adult diabetes patients, the duration of surgery should be planned to minimize fasting and avoid hypoglycemia during the perioperative period [14]. Prolonged fasting increases stress, combined with surgical trauma, leads to increased glucocorticoids and glucagon, reduces insulin sensitivity, and reduces utilization of glucose in peripheral tissues, ultimately leading to postoperative hyperglycemia and increased insulin resistance [15]. Postoperative insulin resistance increases the incidence of hyperglycemia and infection. Carbohydrate intake 2 3 hours before surgery shortens the duration of fasting, increases the energy supply, and reduces perioperative hypoglycemia and insulin resistance.

Most studies of ERAS protocols in general surgery exclude diabetes patients because of concerns of slow gastric emptying and impaired preoperative blood glucose control. Consequently, evidence supporting preoperative carbohydrate intake by diabetes is lacking. Breuer et al. evaluated the impact of 400 ml of a 12.5% carbohydrate beverage 2 hours before heart surgery in ASA status III-IV patients. The carbohydrate did not cause an increase in the volume of gastric juice and was not associated with other adverse events [7]. In Gustafsson et al., type 2 diabetes patients, given 400 ml of a 12.5% carbohydrate beverage, experienced a peak glucose concentration of 13.4 ± 0.5 mm after 60 minutes. The concentration returned to baseline 180 minutes later, and gastric emptying was slightly faster than that in healthy subjects [8]. Although the patients did not undergo surgery, there were no signs of delayed gastric emptying, indicating that carbohydrate drinks could be given safely 180 minutes before anesthesia without the risk of hyperglycemia or preoperative aspiration. Consistent with those results, Laffin et al. [11] reported that 500 ml of cranberry cocktail or apple juice orally 1 hour before bedtime on the night before surgery and again 3 hours before surgery did not increase the incidence of preoperative hyperglycemia. The American Diabetes Association recommends that perioperative blood glucose is to be maintained between 4.4 and 10.0 mmol/L [16]. Gustafsson et al. [8] and Laffin et al. [11] both showed that preoperative carbohydrate intake did not adversely disturb glucose control and potentially reduced the occurrence of adverse reactions such as impaired blood sugar control, delayed gastric emptying, and aspiration. Laffin et al. reported that there were no cases with cancelled surgery because of oral carbohydrates before surgery [11]. The overall evidence of the reviewed studies indicates that preoperative carbohydrate intake did not lead to the cancellation of diabetic surgery or affected the length of hospital stay, pneumonia, and other complications [7, 9, 11].

This qualitative review supports the effectiveness and safety of ERAS protocols with oral carbohydrates for diabetes patients and provides a rationale for the design of future clinical investigations. The conclusions are limited by the inclusion of only five studies. Four studies included only diabetes patients; one included subjects with diabetes and other diseases. Inclusion bias may have affected the results of four studies. Only one study was randomized and double-blinded, and had intervention, placebo, and healthy control arms. The other studies included controls but were not randomized or blinded. The overall study quality was low, and the experimental design and evaluation criteria differ, which did not permit quantitative analysis.

## 5. Conclusion

Inconsistencies exist in the reported effects of carbohydrate intake 2-3 hours before surgery on insulin resistance in diabetes patients, but the available results show a tendency to improve insulin resistance and avoid postoperative hyperglycemia after surgery. Preoperative oral carbohydrates are effective and safe for surgical patients with diabetes mellitus. High-quality randomized controlled trials are needed to provide stronger evidence to support the use of oral carbohydrates in ERAS protocols for diabetes patients.

## Figures and Tables

**Table 1:** Characteristics of the studies included in the systemic review.

First author, (reference) year, region	Preoperative oral carbohydrate type, time, and quantity	Study population				Outcome indicators	Statistical results	Adverse reactions and safety
		Combined disease	Sample size		Age (year)			
			Intervention group	Control group				

Breuer et al. [7], 2006, Germany	Oral 400 ml 12.5% carbohydrate beverage 2 hours before surgery	ASA III–IV heart surgery	56	60	64 ± 9 vs 64 ± 10	Preoperative discomfort VAS score, PIR, length of hospital stay, number of deaths during hospital stay	Preoperative discomfort VAS score were all *P* > 0.05; PIR was *P* > 0.05; length of hospital stay was *P* > 0.05	No increase in gastric juice volume or other adverse events were observed; one patient in the placebo group died of intestinal cancer in the ward during hospital stay

Gustafsson et al. [8], 2008, Sweden	Oral 400 ml 12.5% carbohydrate-rich beverages	NR	25	10	45–73	The peak blood sugar, the time to reach the peak, the time to restore the baseline glucose concentration, and the time of gastric half emptying	The peak blood sugar after beverage intake of diabetic patients vs health population was 13.4 ± 0.5 vs 7.6 ± 0.5 mM, *P* < 0.05; the baseline level restore time was 180 vs 120 min, *P* < 0.05; the half emptying time of the stomach was 49.8 ± 2.2 vs 58.6 ± 3.7 min, *P* < 0.05	No hyperglycemia or aspiration occurred, others were not mentioned

Lu et al. [9], 2015, China	Oral 5% glucose solution 200 ml 2-3 hours before operation	Liver cancer	60	60	56.82 ± 8.23	Thirst, hunger, and anxiety before operation, blood sugar before anesthesia, PIRI complications and hospitalization days after operation	Preoperative thirst/hunger, *P* < 0.05; preoperative anxiety, *P* > 0.05; blood sugar before anesthesia 8.16 ± 2.20 vs 7.48 ± 2.80 mmol/L, *P* > 0.05; PIRI, 3.24 ± 1.07 vs 7.40 ± 3.25, *P* < 0.05; hospitalization days after operation 9.46 ± 4.57 vs 10.03 ± 3.46 *P* > 0.05	Incidence of postoperative complication, *P* > 0.05

He et al. [10], 2017, China	Oral 5% glucose 250 ml 2 hours before operation	Colorectal cancer	64	60	62.3 ± 9.8	FBG on in the morning of operation and on the first day after operation	Operation day morning FBG 6.2 ± 0.5 vs 6.0 ± 0.7, *P* > 0.05; the first day after operation FBG 7.5 ± 2.4 vs 9.3 ± 2.6, *P* < 0.05	NR

Laffin et al. [11], 2018, Canada	Oral 500 ml cranberry cocktail or apple juice 1 hour before bedtime, one night before operation, and 3 hours before operation	Cardiac, neurological, extraurological, and general surgery	46	60	61.8 vs 66.4	Preoperative blood sugar, preoperative incidence of hyperglycemia, length of hospital stay, number of cases of cancelled surgery, incidence of pneumonia after operation, number of deaths in 30 days	The preoperative blood glucose was 8.3 vs 8.1 mmol/L, *P* > 0.05; the preoperative incidence of hyperglycemia was 17.4% vs 16.7%, *P* > 0.05; the number of cancelled operations was 0; and the hospital stay was 0.48	Incidence of pneumonia after operation was *P*=0.58, and no death occurred within 30 days after operation

ASA III-IV, American Society of Anesthesiologists; FBG, fasting blood glucose; NR, not reported; PIR, postoperative insulin resistance; PIRI: postoperative insulin resistance index; VAS, visual analog scale.
